# Radiodensity as a predictor: can penile and splenic Hounsfield units forecast clinical response to daily tadalafil?

**DOI:** 10.1186/s12610-026-00302-8

**Published:** 2026-02-11

**Authors:** Huseyin Hayit, Cumhur Yesildal, Omer Yilmaz, Yahya Barac, Kerem Bursali, Anil Yilmaz

**Affiliations:** 1 University of Health and Science Sultan Abdulhamid Han Training and Research Hospital, Istanbul, Turkey; 2 Cizre Dr.Selahattin Cizrelioglu State Hospital, Sirnak, Turkey

**Keywords:** Erectile dysfunction, Computed tomography, Tissue density, Tadalafil, Microvascular perfusion, Dysfonction érectile, Tomodensitométrie, Densité tissulaire, Tadalafil, Perfusion microvasculaire

## Abstract

**Background:**

Objective imaging biomarkers reflecting penile microvascular perfusion in erectile dysfunction are limited. Hounsfield unit measurements obtained from non-contrast computed tomography may offer a non-invasive approach to assess tissue perfusion. This retrospective cohort study evaluated the associations between penile and splenic tissue density values and erectile function scores, and explored their relationship with response to daily tadalafil therapy.

**Results:**

A total of 194 men with erectile dysfunction who underwent non-contrast lower abdominal computed tomography were included. Tissue density values were measured in proximal, midshaft, and distal penile segments as well as in splenic parenchyma. Daily tadalafil 5 mg was associated with statistically significant improvements across erectile function domains (mean change in erectile function score: +7.9 points; p < 0.0001). Baseline penile tissue density values demonstrated positive correlations with splenic tissue density, suggesting a potential association related to perfusion characteristics. Although responders exhibited lower baseline tissue density values in selected penile segments compared with non-responders, these parameters did not independently predict treatment response in multivariable analysis.

**Conclusions:**

Computed tomography–derived tissue density measurements of penile and splenic tissues may offer complementary, objective information related to tissue perfusion in men with erectile dysfunction. While these findings support the exploratory use of imaging-based density analysis in understanding vascular and metabolic influences on erectile function, prospective multicentre studies with standardized protocols are required to validate their clinical utility.

## Résumé

**Contexte:**

Les biomarqueurs d’imagerie objective reflétant la perfusion microvasculaire pénienne dans la dysfonction érectile sont limités. Les mesures unitaires de Hounsfield, obtenues à partir de tomodensitométrie sans contraste, peuvent offrir une approche non invasive pour évaluer la perfusion tissulaire. Cette étude de cohorte rétrospective a évalué les associations entre les valeurs de densité pénienne et de densité des tissus spléniques avec les scores de fonction érectile ; et elle a exploré leur relation avec la réponse à une thérapie quotidienne par tadalafil.

**Résultats:**

Un total de 194 hommes, souffrant d’une dysfonction érectile et ayant subi une tomodensitométrie sans contraste du bas de l’abdomen, ont été inclus. Les valeurs de densité tissulaire ont été mesurées dans les segments proximal, médian et distal du pénis ainsi que dans le parenchyme splénique. Un traitement quotidien par Tadalafil 5 mg a été associé à des améliorations statistiquement significatives dans les domaines de la fonction érectile (variation moyenne du score de fonction érectile : +7,9 points ; p < 0,0001). Les valeurs de base de la densité du tissu pénien ont montré des corrélations positives avec la densité du tissu splénique, suggérant une association potentielle liée aux caractéristiques de perfusion. Bien que les hommes répondeurs aient présenté des valeurs de densité tissulaire de base plus faibles dans certains segments péniens comparés aux non-répondeurs, ces paramètres n’ont pas prédit de manière indépendante la réponse au traitement dans l’analyse multivariée.

**Conclusions:**

Les mesures de densité tissulaire dérivées de la tomodensitométrie des tissus péniens et splénique peuvent fournir des informations complémentaires et objectives concernant la perfusion tissulaire chez les hommes souffrant de dysfonction érectile. Bien que ces résultats soutiennent l’utilisation exploratoire de l’analyse de densité basée sur l’imagerie pour comprendre les influences vasculaires et métaboliques sur la fonction érectile, des études prospectives multicentriques avec des protocoles standardisés sont nécessaires pour valider leur utilité clinique.

## Introduction

Erectile dysfunction (ED) is a common condition in men, characterized by the inability to achieve or maintain a penile erection, and it significantly impacts quality of life. The etiology of ED is multifactorial, involving vascular, neurogenic, hormonal, anatomical, and psychological factors. Among these, the vascular component is particularly prevalent in older individuals and is often linked to hemodynamic disturbances such as arterial insufficiency or venous leakage [1].

Despite extensive research on erectile dysfunction, current diagnostic approaches largely rely on subjective questionnaires and functional imaging techniques that are operator dependent and limited in their ability to provide quantitative, tissue-level information. To date, there is no widely accepted, objective imaging biomarker derived from routinely available cross-sectional imaging that reflects penile microvascular characteristics and can be evaluated alongside systemic vascular status.

Computed tomography allows standardized, quantitative assessment of tissue density through Hounsfield unit measurements and is frequently performed for various clinical indications. While no validated imaging biomarker currently exists to reflect penile microvascular perfusion in erectile dysfunction, tissue density analysis may provide indirect insight into local vascular characteristics. The spleen was included as an adjunctive parameter based on its rich vascularization and prior use in radiological studies as a pragmatic surrogate of systemic perfusion [2, 3].

Tadalafil, a selective phosphodiesterase type 5 inhibitor (PDE5i), enhances erectile function by potentiating the nitric oxide cyclic guanosine monophosphate pathway, resulting in smooth muscle relaxation and improved penile blood flow [4]. Although PDE5i therapy is well established for symptom relief in ED, its effects on CT-derived HU values in penile tissue remains unexplored.

The aim of this study was to investigate the relationship between HU values obtained from different anatomical regions of the penile tissue and splenic HU values in patients with ED, as well as to assess changes in HU values following tadalafil treatment. This approach aims to determine whether CT-based HU analysis could serve as a potential biomarker for understanding the pathophysiology of ED and evaluating treatment response.

## Patients and methods

### Study design and population

Following approval from the institutional ethics committee, a retrospective review was conducted of patients who presented to the urology outpatient clinic at our hospital between January 1, 2018, and December 31, 2020. Patients diagnosed with ED were identified, and their records were reviewed from the institutional archives.

Patients were included if they met the following criteria:

- Completion of the International Index of Erectile Function (IIEF) questionnaire.
- Received tadalafil at a dose of 5 mg once daily for one month.
- Underwent non-contrast lower abdominal CT imaging both before and after treatment for any clinical reason, with complete visualization of the penis on the scan, and.
- Returned for follow-up and completed a post-treatment IIEF questionnaire.

**Fig. 1:**
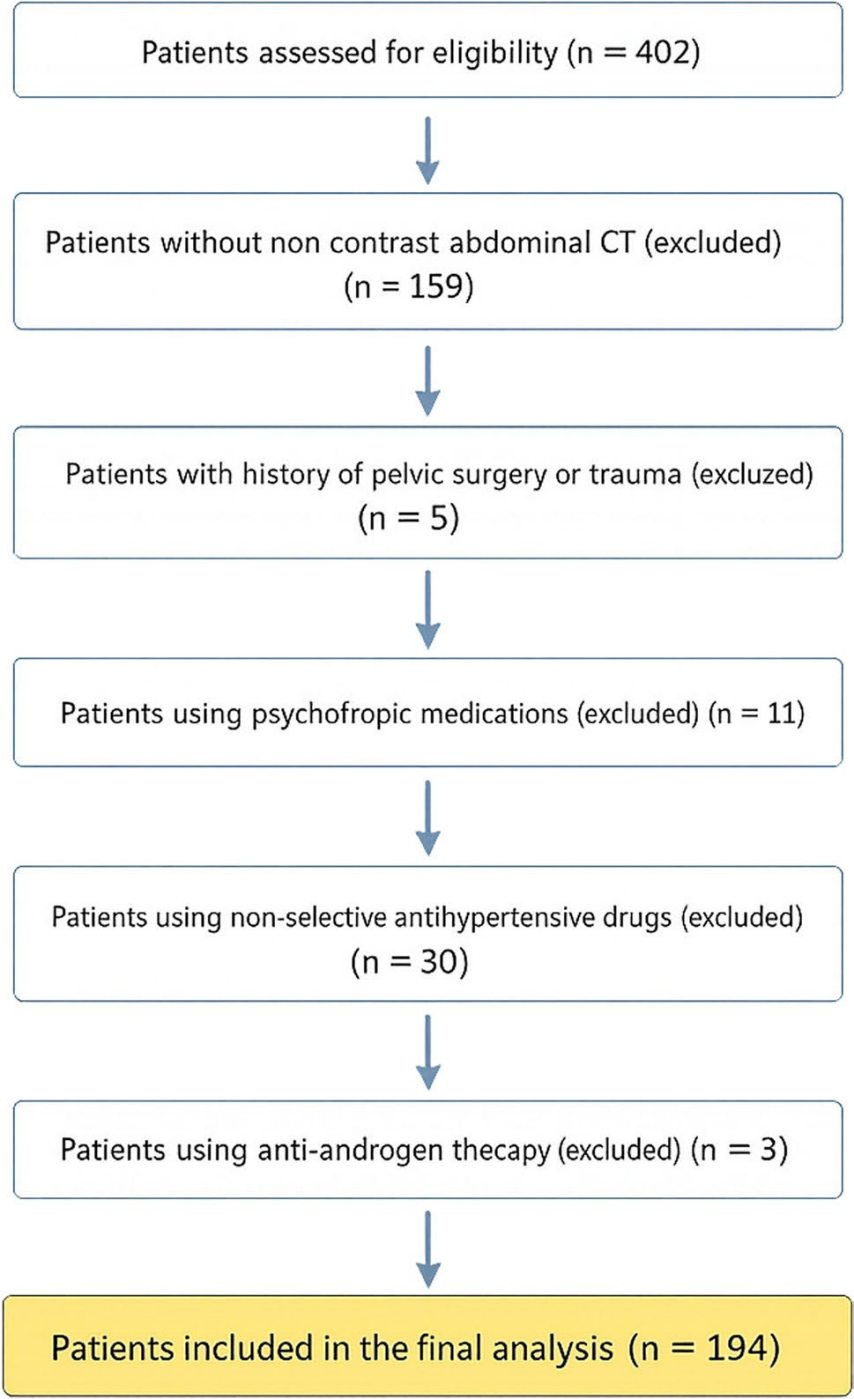
Study Flow Chart: Flow diagram of the study population. A total of 402 patients were screened. Patients without non-contrast computed tomography imaging, those with a history of pelvic surgery or trauma, and patients receiving psychotropic, non-selective antihypertensive, or anti-androgen medications were excluded. After applying the exclusion criteria, 194 patients were included in the final analysis

Exclusion criteria included a history of penile or pelvic surgery, pelvic trauma, or malignancy, as these conditions may directly alter penile anatomy, vascular integrity, or erectile physiology. Patients receiving anti-androgen therapy or non-selective antihypertensive medications were excluded due to their known effects on erectile function. In addition, patients using psychiatric medications known to impair sexual function—including antidepressants, antipsychotics, mood stabilizers, and anxiolytic agents—were excluded to minimize pharmacological confounding. These exclusion criteria were applied to reduce heterogeneity and to isolate vascular and tissue-related factors influencing erectile function and response to tadalafil therapy. The process of patient selection, exclusion, and final cohort formation is summarized in the study flow chart (Fig. 1).

### Imaging protocol and HU measurement

CT images were gained on a 128-slice scanner (Model XYZ; Manufacturer) using standardized settings (120 kVp, auto-mA, 1 mm slice thickness). Although some patients initially presented with isolated flank pain, repeated non-contrast CT examinations were performed for routine clinical indications during follow-up, including recurrent symptoms, known or suspected urolithiasis, hematuria, or hydronephrosis. No CT scan was obtained for the evaluation of erectile dysfunction, and all imaging was performed as part of standard clinical care. Two independent radiologists—blinded to all clinical information—manually placed regions of interest (ROIs) for HU measurement. Since there is no standardized protocol for measuring penile HU values in the literature, measurements were taken at the distal penis (near the glans), midshaft, and proximal region (near the penile root) on both the right and left corpora cavernosa. To improve accuracy, average HU values from the right and left sides were calculated and grouped accordingly. The spleen was included as an adjunctive parameter in the assessment of perfusion-related characteristics, based on prior radiological observations linking splenic imaging features to systemic hypoperfusion [2, 3].

- Penile segments: Three circular ROIs (5 mm² each) were placed in the proximal, midshaft, and distal corpora cavernosa on axial slices exhibiting maximal cross-sectional area.
- Splenic parenchyma: A single ROI (10 mm²) was placed in the mid-spleen, avoiding vessels and artifacts.
- Mean HU values for each ROI were recorded and averaged between observers; inter-observer agreement was assessed with intra-class correlation coefficients (ICC).

Additional data were obtained from hospital records, including patient age, smoking status, alcohol use, comorbidities, surgical history, current medications, pre- and post-treatment IIEF scores, and laboratory parameters. These parameters included total testosterone (TT), prolactin, follicle-stimulating hormone (FSH), luteinizing hormone (LH), thyroid-stimulating hormone (TSH), prostate-specific antigen (PSA), fasting blood glucose (FBG), and hemoglobin A1c (HbA1c) levels.

### Laboratory measurements

Biochemical and hormonal parameters were obtained from institutional laboratory records.

Serum total testosterone, prolactin, follicle-stimulating hormone, luteinizing hormone, thyroid-stimulating hormone, and prostate-specific antigen levels were measured using automated chemiluminescent immunoassay methods in routine clinical practice [5–7].

Fasting blood glucose levels were determined using an enzymatic colorimetric method [8], and glycated hemoglobin (HbA1c) levels were measured using high-performance liquid chromatography [9].

All laboratory analyses were performed in the hospital’s central laboratory according to standard operating procedures and manufacturer instructions, in accordance with established clinical guidelines.

The validated Turkish version of the International Index of Erectile Function questionnaire was used for assessment of erectile dysfunction [10]. The IIEF questionnaire responses were categorized into five subdomains:

- Erectile function (questions 1–5 and 15).
- Sexual satisfaction (questions 6–8).
- Orgasmic function (questions 9–10).
- Sexual desire (questions 11–12), and.
- Overall satisfaction (questions 13–14).

Symptomatic changes were monitored following tadalafil treatment. Patients were divided into two groups: pre-treatment (Group A) and post-treatment (Group B). Treatment response was evaluated by comparing IIEF scores before and after treatment. A significant improvement in scores was considered indicative of a treatment response.

### Statistical analysis

Continuous variables are expressed as mean ± SD or median (IQR). Paired t-tests compared IIEF scores before and after treatment. Pearson correlation coefficients assessed relationships between HU values, IIEF domains, and metabolic parameters (fasting glucose, HbA1c). Independent-samples t-tests compared baseline HU values between responders and non-responders. Inter-observer reliability was evaluated using ICC, with values > 0.75 indicating excellent agreement. Statistical significance was set at *p* < 0.05 (two-sided). In this study, paired measurements obtained before and after treatment were compared using the paired two-tailed t-test. The sample size was *N* = 194. A post-hoc compromise power analysis was performed using G*Power version 3.1. Assuming a small effect size (dz = 0.25), the analysis yielded a statistical power (1–β) of 0.89 at a significance level of α = 0.05. All analyses were performed using SPSS v26.0 (IBM Corp., Armonk, NY).

## Results

Following a three-year retrospective file review, a total of 194 male patients with complete clinical data and available non-contrast CT scans were included in the study. These cases were selected from among 402 patients who had presented to the urology outpatient clinic with complaints of ED. The mean age of the included patients was 54.34 ± 9.64 years, the mean body mass index (BMI) was 29.41 ± 4.74 kg/m², other demographic and clinical characteristics of the study population are summarized in Table 1.Clinical profile findings, including nocturnal penile tumescence(NPT) status, libido, and comorbid conditions such as diabetes mellitus(DM) and hypertension(HT), are presented in Table 2.

**Table 1:** Distribution of demographic characteristics (*n* = 194)

Parameter	Mean ± SD	Median	Min-Max
Height (cm)	172.57 ± 14.45	173.00	158–192
Age	54.34 ± 9.64	55.00	26–73
Weight	88.17 ± 12.77	86.00	53–140
BMI	29.41 ± 4.74	28.73	19.41–65.65
Duration of ED (months)	35.64 ± 40.68	24.00	1–240

This table summarizes the demographic characteristics of the study population. Continuous variables are presented as mean ± standard deviation (SD) and median (minimum–maximum). Body mass index (BMI) was calculated as weight divided by height squared (kg/m²)

BMI Body mass index, SD Standard deviation

**Table 2:** Distribution of participants by NPT, Libido, DM, and HT status (*n* = 194)

Category	Status	*n*	%
NPT	Absent	120	61.8%
NPT	Present	74	38.2%
Libido	Absent	59	30.5%
Libido	Present	135	69.5%
DM	Absent	143	73.7%
DM	Present	51	26.3%
HT	Absent	140	72.2%
HT	Present	54	27.8%

This table presents the distribution of key clinical characteristics of the participants, including nocturnal penile tumescence, libido status, diabetes mellitus, and hypertension. Data are expressed as number (n) and percentage (%)

*DM* Diabetes mellitus,* HT* Hypertension,* NPT* Nocturnal penile tumescence

**Fig. 2:**
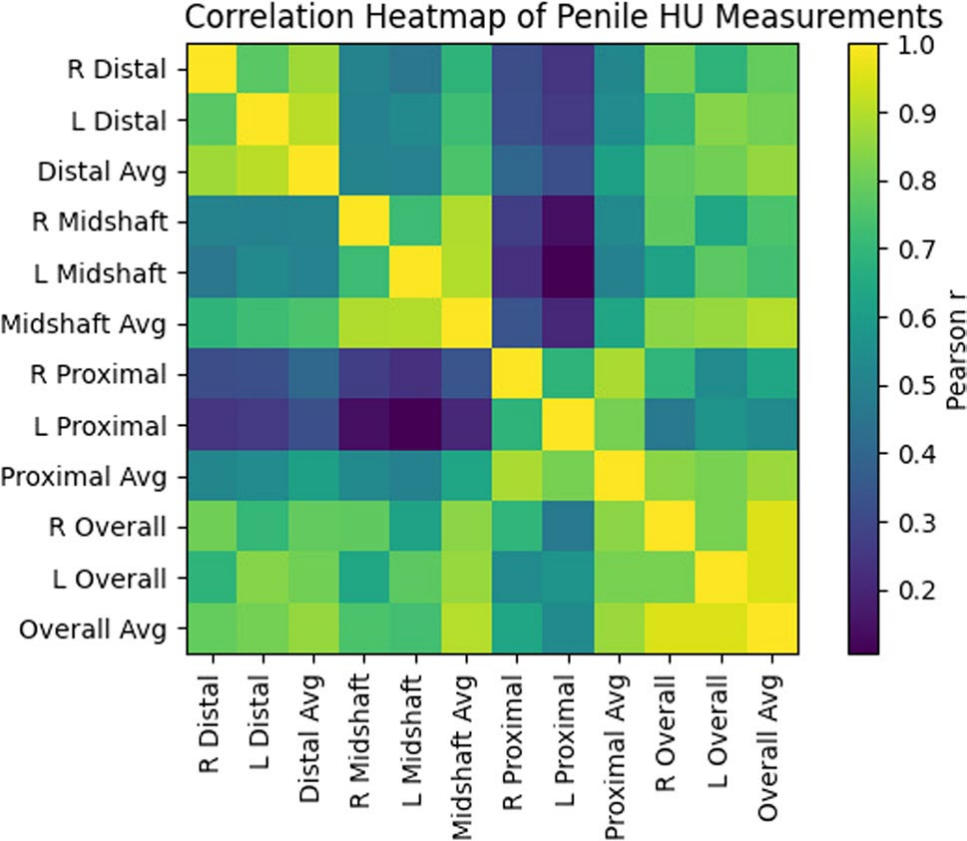
Correlation heatmap of Hounsfield unit values across penile segments: This heatmap illustrates Pearson’s correlation coefficients (r) between Hounsfield unit (HU) values measured across different penile segments. Color intensity reflects the strength and direction of the correlations, with darker colors indicating stronger associations. The figure provides a visual summary of inter-segmental consistency of HU measurements. Abbreviations: Avg, average; HU, Hounsfield unit; L, left; R, right

Biochemical parameters are summarized in Table 3.Among the patients, 81 underwent non-contrast CT imaging for evaluation of flank pain, 55 for known upper urinary tract calculi, and 2 for suspected urethral stones. The full distribution of CT indications is presented in Table 4.HU values measured from the spleen and various penile segments is presented in Table 5.In the questionnaire-based assessment, a statistically significant positive correlation was observed between erectile function scores and the left midshaft penile HU value (*p* < 0.05) (Table 6).In the post-treatment questionnaire assessment, a statistically significant negative correlation was identified between sexual satisfaction scores and the following HU values: right distal penile HU, right midshaft penile HU, midshaft penile average HU, and right overall penile HU (*p* < 0.05) (Table 7).A statistically significant positive correlation was observed between splenic HU values and the following penile HU measurements: right distal, left distal, distal average, midshaft average, right proximal, left proximal, proximal average, right overall, left overall, and overall penile HU (*p* < 0.05) (Table 8).**….**When evaluating the correlations between HU values across different penile segments, no statistically significant association was found between the left midshaft and left proximal HU values (*p* > 0.05). All other segmental comparisons demonstrated statistically significant correlations (*p* < 0.05) Inter-segmental correlations are visually summarized in Fig. 2.Evaluation of pre- and post-treatment IIEF scores revealed statistically significant improvements across all domains—including erectile function, sexual satisfaction, orgasmic function, sexual desire, and overall satisfaction—following one month of daily 5 mg tadalafil treatment (*p* = 0.0001). A summary of the IIEF scores before and after treatment is provided in Table 9.Following tadalafil treatment, each IIEF domain was evaluated separately. Patients who showed no increase in domain-specific scores compared to baseline were categorized as Group 1, while those with improved scores were categorized as Group 2. Using splenic HU as a reference, comparisons of penile HU values between the two groups revealed that the left midshaft penile HU was significantly lower in the treatment-responsive group based on erectile function scores (*p* = 0.04). In contrast, for orgasmic function, the left proximal penile HU was significantly higher in the responsive group (*p* = 0.04). No other significant differences in penile HU values were observed between groups (*p* > 0.05). Comparative data are presented in Tables 10 and 11.A statistically significant negative correlation was observed between FBG and splenic HU values (*p* < 0.05), indicating a decrease in splenic perfusion with rising glucose levels.

**Table 3:** Distribution of biochemical parameters (*n* = 194)

Parameter	Mean ± SD	Median	Min-Max
FBG	112.95 ± 41.84	102	51–350
PSA	21.01 ± 229.18	1.17	0.01–3090
TT	4.66 ± 3.15	3.99	1.36–26.29
PRL	10.71 ± 6.04	9.2	2.6–40.74.6.74
FSH	6.41 ± 4.71	5.1	1.41–27.35
LH	5.13 ± 2.99	4.14	1.4–22.4
TSH	1.71 ± 1.17	1.37	0.01–6.54
HbA1c	6.27 ± 1.44	6	0–14.2.2

This table shows baseline biochemical and hormonal parameters of the study population. Values are reported as mean ± standard deviation and median (minimum–maximum). Laboratory measurements were obtained from hospital records at baseline

*FBG* Fasting blood glucose, *FSH* Follicle-stimulating hormone, *HbA1c* Hemoglobin A1c,* LH* Luteinizing hormone,* PRL* Prolactin, *PSA* Prostate-specific antigen, *SD* Standard deviation, *TSH* Thyroid-stimulating hormone, *TT* Total testosterone

**Table 4:** Indications for Non-Contrast CT scans in patients

Indication for Scan	*n*	%
Flank pain	81	41.8%
Upper urinary tract stone	55	28.4%
Hematuria	47	24.2%
Hydronephrosis	9	4.6%
Urethral stone	2	1%
Total	194	100%

This table lists the clinical indications for which non-contrast computed tomography (CT) scans were performed in the study population. Data are expressed as number (n) and percentage (%)

**Table 5:** Distribution by HU values (*n* = 194)

Parameter	Mean ± SD	Median	Min-Max
Splenic HU	50.76 ± 3.35	51	42–61
Right Distal Penile HU	44.79 ± 4.55	44	36–58
Left Distal Penile HU	45.16 ± 4.80	44	37–60
Distal Penile Average HU	44.98 ± 4.68	44	36.5–59
Right Midshaft Penile HU	47.38 ± 4.48	47	39–59
Left Midshaft Penile HU	47.82 ± 4.73	47	40–61
Midshaft Penile Average HU	47.40 ± 3.59	46.78	40.67–58.89
Right Proximal Penile HU	49.35 ± 4.42	49	37–59
Left Proximal Penile HU	49.46 ± 3.89	49	39–60
Proximal Penile Average HU	49.41 ± 4.16	49	38–59.5
Right Overall Penile HU	47.17 ± 3.40	46.67	40.33–58.33
Left Overall Penile HU	47.49 ± 3.29	47	41.67–59.67
Overall Penile Average HU	47.47 ± 3.11	46.87	41.62–58.65

This table presents Hounsfield unit (HU) values measured from the spleen and different penile segments on non-contrast CT images. Values are reported as mean ± standard deviation and median (minimum–maximum). Average HU values represent the arithmetic mean of the right and left measurements for the corresponding penile segment

*HU* Hounsfield unit,* SD* Standard deviation

**Table 6:** Analysis of the relationship between Pre-Tadalafil treatment questionnaire results and HU values

Parameter	Erectile Function	Orgasmic Function	Sexual Desire	Sexual Satisfaction	Overall Satisfaction
Splenic HU	0.041	0.07	0.022	0.035	0.003
Right Distal Penile HU	−0.027	0.011	−0.022	0.029	−0.062
Left Distal Penile HU	0.044	0.027	−0.037	0.078	0.018
Distal Penile Average HU	0.011	0.026	−0.023	0.047	−0.029
Right Midshaft Penile HU	0.008	0.069	−0.054	0.037	0.024
Left Midshaft Penile HU	0.171^a^	0.126	−0.012	0.138	0.148
Midshaft Penile Average HU	0.075	0.083	−0.037	0.082	0.058
Right Proximal Penile HU	0.042	0.08	0.029	0.072	0.03
Left Proximal Penile HU	−0.031	0.026	0.021	−0.015	−0.015
Proximal Penile Average HU	0.031	0.074	0.007	0.053	0.026
Right Overall Penile HU	0.01	0.07	−0.021	0.06	−0.004
Left Overall Penile HU	0.091	0.083	−0.015	0.098	0.074
Overall Penile Average HU	0.042	0.066	−0.022	0.068	0.023

This table shows the correlations between baseline International Index of Erectile Function (IIEF) domain scores and HU values measured from the spleen and penile segments before tadalafil treatment. Values represent Pearson’s correlation coefficients (r)

Analyses were performed in 194 patients unless otherwise specified

*HU* Hounsfield unit

^a^*p* < 0.05 statistically significant

**Table 7:** Analysis of the relationship between Post-Tadalafil treatment questionnaire results and HU values

Parameter	Erectile Function	Orgasmic Function	Sexual Desire	Sexual Satisfaction	Overall Satisfaction
Splenic HU	0.037	0.018	0.059	−0.052	0.118
Right Distal Penile HU	−0.22	−0.042	−0.091	−0.275^a^	−0.108
Left Distal Penile HU	−0.037	0.088	−0.051	−0.081	0.066
Distal Penile Average HU	−0.107	0.026	−0.045	−0.185	0.02
Right Midshaft Penile HU	−0.147	−0.095	−0.148	−0.273^a^	−0.089
Left Midshaft Penile HU	−0.108	−0.179	−0.153	−0.183	−0.05
Midshaft Penile Average HU	−0.145	−0.115	−0.147	−0.257^a^	−0.055
Right Proximal Penile HU	0.138	0.2	0.021	0.031	0.193
Left Proximal Penile HU	0.011	0.155	0.028	−0.009	0.061
Proximal Penile Average HU	0.011	0.116	−0.04	−0.098	0.098
Right Overall Penile HU	−0.114	0.02	−0.103	−0.245^a^	−0.01
Left Overall Penile HU	−0.074	0.007	−0.099	−0.146	0.029
Overall Penile Average HU	−0.1	0.01	−0.102	−0.21	0.012

This table demonstrates correlations between post-treatment IIEF domain scores and HU values of the spleen and penile segments following one month of tadalafil therapy. Values represent Pearson’s correlation coefficients (r)

Analyses were performed in 194 patients unless otherwise specified

HU = Hounsfield unit

^a^ *p* < 0.05 statistically significant

**Table 8:** Analysis of the relationship between Splenic HU and penile HU values

HU Correlation with Splenic HU	Correlation Coefficient	Statistically Significant (*p* < 0.05)
Right Distal Penile HU	0.17	^a^
Left Distal Penile HU	0.253	^a^
Distal Penile Average HU	0.526	^a^
Right Midshaft Penile HU	0.128	
Left Midshaft Penile HU	0.143	
Midshaft Penile Average HU	0.282	^a^
Right Proximal Penile HU	0.329	^a^
Left Proximal Penile HU	0.287	^a^
Proximal Penile Average HU	0.383	^a^
Right Overall Penile HU	0.273	^a^
Left Overall Penile HU	0.303	^a^
Overall Penile Average HU	0.35	^a^

This table presents the correlations between splenic HU values and HU measurements obtained from different penile segments. Correlation coefficients were calculated using Pearson’s correlation analysis

Analyses were performed in 194 patients unless otherwise specified

*HU* Hounsfield unit

^a^*p* < 0.05 statistically significant

**Table 9:** IIEF scores before and after one month of Tadalafil 5 mg treatment and their comparison (*n* = 194)

Domain	Pre-Treatment (Mean ± SD)	Post-Treatment (Mean ± SD)	*p*-value
Erectile Function	15.78 ± 8.24	23.27 ± 6.07	0.0001
Sexual Satisfaction	6.43 ± 3.89	10.06 ± 3.15	0.0001
Orgasmic Function	5.96 ± 3.3	8.11 ± 2.15	0.0001
Sexual Desire	6.02 ± 2.26	7.31 ± 1.54	0.0001
Overall Satisfaction	5.54 ± 2.4	7.59 ± 1.89	0.0001

This table compares International Index of Erectile Function (IIEF) domain scores before and after one month of daily tadalafil 5 mg treatment. Values are expressed as mean ± standard deviation. Pre- and post-treatment comparisons were performed using paired t tests

*IIEF* International Index of Erectile Function,* SD* Standard deviation

**Table 10:** Evaluation of the determining role of penile HU in treatment Response – 1

Parameter	EF Group 1 (Mean ± SD) (*n* = 8)	EF Group 2 (Mean ± SD) (*n* = 63)	EF *p*-value	SS Group 1 (Mean ± SD) (*n* = 13)	SS Group 2 (Mean ± SD) (*n* = 58)	SS *p*-value
Splenic HU	50.38 ± 4.44	50.72 ± 3.42	0.83	50.25 ± 4.05	50.77 ± 3.43	0.50
Right Distal Penile HU	47.38 ± 5.6	44.53 ± 4.45	0.20	46.17 ± 6.44	44.59 ± 4.19	0.85
Left Distal Penile HU	45.13 ± 4.42	44.95 ± 4.41	0.89	44.75 ± 5.5	45.02 ± 4.16	0.49
Distal Penile Average HU	47.63 ± 4.11	46.73 ± 3.19	0.64	47.06 ± 4.41	46.79 ± 3.04	0.82
Right Midshaft Penile HU	47.38 ± 4.63	47.23 ± 4.64	0.92	48.17 ± 5.22	47.05 ± 4.49	0.62
Left Midshaft Penile HU	51.38 ± 4.63	47.62 ± 4.89	0.04*	49.33 ± 4.52	47.79 ± 5.07	0.23
Midshaft Penile Average HU	48.79 ± 4.02	47.19 ± 3.53	0.27	48.19 ± 4.33	47.21 ± 3.44	0.59
Right Proximal Penile HU	49.88 ± 6.49	49.25 ± 3.86	0.95	49.08 ± 5.11	49.38 ± 4.02	0.76
Left Proximal Penile HU	49.13 ± 3.91	49.05 ± 3.62	0.91	49.75 ± 2.99	48.91 ± 3.75	0.39
Proximal Penile Average HU	49.26 ± 3.76	48.5 ± 2.71	0.67	49.01 ± 3.61	48.5 ± 2.67	0.96
Right Overall Penile HU	48.21 ± 4.26	47.01 ± 3.15	0.52	47.81 ± 4.86	47.01 ± 2.88	0.88
Left Overall Penile HU	48.54 ± 3.48	47.21 ± 2.9	0.30	47.94 ± 3.66	47.24 ± 2.83	0.77
Overall Penile Average HU	48.44 ± 3.7	47.23 ± 2.8	0.44	47.94 ± 4.07	47.25 ± 2.64	0.99

This table compares penile and splenic HU values between patients with and without improvement in erectile function and sexual satisfaction following tadalafil treatment. Comparisons between groups were performed using independent samples t tests. Group 1 represents patients without improvement in domain-specific IIEF scores, whereas Group 2 represents patients with improvement after tadalafil treatment

*EF* Erectile function,* HU* Hounsfield unit, *SD* Standard deviation,* SS* Sexual satisfaction

**Table 11:** Evaluation of the determining role of penile HU in treatment Response – 2

Parameter	Orgasmic Function Group 1 (Mean ± SD) (*n* = 19)	Orgasmic Function Group 2 (Mean ± SD) (*n* = 52)	Orgasmic Function *p*-value	Sexual Desire Group 1 (Mean ± SD) (*n* = 23)	Sexual Desire Group 2 (Mean ± SD) (*n* = 48)	Sexual Desire *p*-value	Overall Satisfaction Group 1 (Mean ± SD) (*n*=)	Overall Satisfaction Group 2 (Mean ± SD) (*n*=)	Overall Satisfaction *p*-value
Splenic HU	50.84 ± 3.73(51)	50.61 ± 3.47(51)	0.93	51 ± 4.01(51)	50.52 ± 3.29(51)	0.85	50 ± 3.55(50)	50.98 ± 3.5(51)	0.22
Right Distal Penile HU	45.37 ± 4.61(44)	44.67 ± 4.7(43)	0.55	45.73 ± 4.5(45)	44.46 ± 4.71(43)	0.21	44.52 ± 4.15(43)	45.02 ± 4.89(44)	0.83
Left Distal Penile HU	44.63 ± 4.09(43)	45.1 ± 4.52(44)	0.74	45.14 ± 3.89(45)	44.89 ± 4.63(43)	0.58	44.29 ± 3.98(43)	45.28 ± 4.56(44)	0.38
Distal Penile Average HU	46.95 ± 3.07(46.67)	46.8 ± 3.39(46)	0.73	47.29 ± 3.11(47)	46.62 ± 3.38(45.83)	0.29	46.27 ± 3.04(46)	47.09 ± 3.39(46.67)	0.33
Right Midshaft Penile HU	46.63 ± 4.25(47)	47.49 ± 4.76(46)	0.61	45.95 ± 4.01(45)	47.87 ± 4.79(47)	0.12	46.52 ± 3.96(45)	47.57 ± 4.88(47)	0.41
Left Midshaft Penile HU	49 ± 5.6(48)	47.69 ± 4.73(47)	0.37	47.77 ± 5.48(47.5)	48.2 ± 4.78(47)	0.73	48.38 ± 5.29(47)	47.91 ± 4.88(47)	0.71
Midshaft Penile Average HU	47.53 ± 3.67(46.67)	47.33 ± 3.6(46.67)	0.89	47.01 ± 3.62(46.5)	47.56 ± 3.61(46.78)	0.47	47.06 ± 3.47(46)	47.53 ± 3.67(46.89)	0.50
Right Proximal Penile HU	48.84 ± 3.86(48)	49.51 ± 4.34(49)	0.54	49.23 ± 3.62(50)	49.37 ± 4.47(48.5)	0.99	49.19 ± 4.04(49)	49.38 ± 4.3(49)	0.80
Left Proximal Penile HU	47.63 ± 3(48)	49.61 ± 3.72(50)	0.04	48.64 ± 2.98(49)	49.26 ± 3.91(49)	0.63	49.52 ± 2.75(49)	48.85 ± 3.96(49)	0.45
Proximal Penile Average HU	48 ± 2.16(47.74)	48.82 ± 3.04(48.37)	0.30	48.29 ± 2(48.24)	48.73 ± 3.16(48.06)	0.94	48.59 ± 2.43(48.26)	48.59 ± 3.02(48.15)	0.85
Right Overall Penile HU	46.95 ± 2.77(46)	47.22 ± 3.48(46.33)	0.83	46.97 ± 2.63(46.33)	47.23 ± 3.58(46.33)	0.98	46.75 ± 2.63(46.33)	47.33 ± 3.55(46.33)	0.76
Left Overall Penile HU	47.09 ± 2.81(46)	47.47 ± 3.05(47)	0.57	47.18 ± 2.61(46.67)	47.45 ± 3.16(47)	0.83	47.4 ± 2.83(46.67)	47.35 ± 3.07(47)	0.98
Overall Penile Average HU	47.18 ± 2.66(45.96)	47.45 ± 3.03(46.75)	0.83	47.23 ± 2.5(46.5)	47.44 ± 3.12(46.85)	0.85	47.15 ± 2.55(46.71)	47.47 ± 3.08(46.97)	0.87

This table compares HU values between responders and non-responders based on orgasmic function, sexual desire, and overall satisfaction domains after tadalafil treatment. Values are presented as mean ± standard deviation (median). Group comparisons were conducted using independent samples t tests

*HU* Hounsfield unit, *SD* Standard deviation

PSA levels demonstrated a positive correlation with several penile HU measurements, including the left distal, distal average, left midshaft, midshaft average, and overall penile average HU (*p* < 0.05).

Similarly, TT levels showed a positive correlation with HU values of the left distal, distal average, left proximal, proximal average, left midshaft, and overall penile average regions (*p* < 0.05).

In contrast, PRL levels were negatively correlated with the right overall penile HU (*p* < 0.05).

A negative correlation was also found between LH and the left distal, distal average, and midshaft average HU values (*p* < 0.05), while TSH levels negatively correlated with the left proximal penile HU (*p* < 0.05).

Finally, HbA1c levels were negatively correlated with splenic HU, distal penile average HU, and midshaft penile average HU (*p* < 0.05).

The correlations between HU values and biochemical parameters are summarized in Table 12.A statistically significant negative correlation was found between age and splenic HU (*p* < 0.05), suggesting a decline in systemic perfusion with advancing age.

**Table 12:** Analysis of the relationship between HU values and biochemical parameters

Splenic HU	FBG	PSA	TT	PRL	FSH	LH	TSH	HbA1c
	−0.164^a^	0.149	0.233^a^	−0.027	−0.022	−0.162	−0.026	−0.208^a^
Right Distal Penile HU	−0.069	0.123	0.117	−0.096	0.032	−0.156	−0.122	−0.107
Left Distal Penile HU	−0.043	0.186^a^	0.17^a^	−0.043	0.042	−0.172^a^	−0.087	−0.124
Distal Penile Average HU	−0.106	0.191^a^	0.238^a^	−0.074	0.03	−0.2^a^	−0.099	−0.17
Right Midshaft Penile HU	−0.009	0.096	0.019	−0.168^a^	−0.088	−0.124	−0.046	−0.12
Left Midshaft Penile HU	−0.001	0.133	0.019	−0.078	0.035	−0.148	−0.01	−0.133
Midshaft Penile Average HU	−0.037	0.158^a^	0.111	−0.129	−0.012	−0.18^a^	−0.053	−0.16^a^
Right Proximal Penile HU	−0.018	0.034	0.097	−0.099	0.05	−0.041	−0.025	−0.05
Left Proximal Penile HU	0.041	0.15	0.183^a^	−0.028	0.068	0.013	−0.166^a^	0.01
Proximal Penile Average HU	−0.006	0.138	0.17^a^	−0.107	0.047	−0.083	−0.099	−0.08
Right Overall Penile HU	−0.042	0.112	0.104	−0.161^a^	−0.002	−0.143	−0.086	−0.121
Left Overall Penile HU	−0.004	0.214^a^	0.163^a^	−0.07	0.066	−0.146	−0.114	−0.119
Overall Penile Average HU	−0.035	0.174^a^	0.178^a^	−0.122	0.03	−0.161	−0.098	−0.134

This table shows correlations between HU values and biochemical or hormonal parameters. Correlation coefficients were calculated using Pearson’s correlation analysis

Analyses were performed in 194 patients unless otherwise specified

*FBG* Fasting blood glucose,* FSH* Follicle-stimulating hormone,* HbA1c* Hemoglobin A1c,* HU* Hounsfield unit,* LH* Luteinizing hormone,* PRL* Prolactin,* PSA* Prostate-specific antigen,* TSH* Thyroid-stimulating hormone,* TT *Total testosterone

^a^*p* < 0.05 statistically significant

BMI demonstrated significant negative correlations with HU values of the spleen, distal penile average, midshaft penile average, left proximal, proximal average, and overall penile average regions (*p* < 0.05).

The duration of ED was also negatively correlated with right distal penile HU (*p* < 0.05).

Additionally, libido was negatively associated with splenic HU (*p* < 0.05), indicating a possible link between systemic perfusion and sexual drive.

Among comorbidities, DM showed statistically significant negative correlations with right midshaft, midshaft average, left overall, and overall penile average HU values (*p* < 0.05).

Likewise, HT was negatively correlated with HU values in the distal average, midshaft average, proximal average, right overall, and overall penile average penile regions (*p* < 0.05).

The relationships between HU values and demographic and clinical parameters are presented in Table 13.When patients were stratified based on total erectile function scores—classified as ≥ 22 versus ≤ 21—no statistically significant associations were found between HU values and treatment response in the higher-scoring group.

**Table 13:** Analysis of the relationship between HU values and study Parameters**

HU Parameter	Age^b^	BMI^b^	Duration of ED (months)^b^	NPT^c^	Libido^c^	DM^c^	HT^c^
Splenic HU	−0.183^a^	−0.294^a^	−0.077	0.026	−0.229^a^	−0.098	−0.125
Right Distal Penile HU	0.006	−0.125	−0.166^a^	0.117	−0.132	−0.113	−0.11
Left Distal Penile HU	−0.004	−0.105	−0.099	0.12	−0.029	−0.121	−0.124
Distal Penile Average HU	−0.049	−0.204^a^	−0.151	0.123	−0.136	−0.145	−0.163^a^
Right Midshaft Penile HU	−0.052	−0.137	−0.149	−0.009	−0.047	−0.202^a^	−0.124
Left Midshaft Penile HU	0.038	−0.147	−0.079	0.162	0.035	−0.141	−0.076
Midshaft Penile Average HU	−0.016	−0.189^a^	−0.144	0.134	−0.025	−0.206^a^	−0.153^a^
Right Proximal Penile HU	−0.066	−0.133	−0.077	−0.009	−0.114	0.005	−0.128
Left Proximal Penile HU	−0.067	−0.167^a^	−0.063	−0.038	0.036	−0.06	−0.107
Proximal Penile Average HU	−0.062	−0.206^a^	−0.119	0.043	−0.06	−0.075	−0.169^a^
Right Overall Penile HU	−0.048	−0.171^a^	−0.175^a^	0.074	−0.082	−0.15	−0.157^a^
Left Overall Penile HU	−0.009	−0.182^a^	−0.113	0.159	0.015	−0.178^a^	−0.142
Overall Penile Average HU	−0.03	−0.203^a^	−0.154	0.151	−0.049	−0.167^a^	−0.168^a^

This table presents correlations between HU values and demographic or clinical parameters, including age, body mass index, duration of erectile dysfunction, nocturnal penile tumescence, libido, diabetes mellitus, and hypertension. Pearson’s correlation was used for continuous variables, and Spearman’s rho was applied for categorical variables

Analyses were performed in 194 patients unless otherwise specified

*BMI* Body mass index,* DM* Diabetes mellitus,* HT* Hypertension,* HU* Hounsfield unit,* NPT* Nocturnal penile tumescence

^a^*p* < 0.05 statistically significant; ^b^Pearson’s correlation coefficient; ^c^Spearman’s rho

To further investigate the predictive value of HU parameters, a logistic regression analysis was conducted. However, none of the HU measurements emerged as statistically significant independent predictors of treatment response (Table 14).

**Table 14:** Logistic regression analysis

Splenic HU	B	*p*	Exp(B)	Lower CI	Upper CI
	0.002	0.973	1.002	0.907	1.106
Right Distal Penile HU	−0.051	0.199	0.95	0.88	1.027
Left Distal Penile HU	−0.023	0.53	0.977	0.91	1.05
Distal Penile Average HU	−0.05	0.337	0.951	0.859	1.054
Right Midshaft Penile HU	−0.057	0.157	0.945	0.874	1.022
Left Midshaft Penile HU	−0.008	0.818	0.992	0.924	1.064
Midshaft Penile Average HU	−0.051	0.303	0.95	0.863	1.047
Right Proximal Penile HU	0.002	0.967	1.002	0.929	1.08
Left Proximal Penile HU	−0.054	0.23	0.948	0.869	1.034
Proximal Penile Average HU	−0.05	0.368	0.951	0.853	1.061
Right Overall Penile HU	−0.062	0.237	0.94	0.847	1.042
Left Overall Penile HU	−0.048	0.37	0.953	0.858	1.059
Overall Penile Average HU	−0.066	0.254	0.936	0.836	1.048

This table presents the results of logistic regression analysis evaluating HU parameters as potential independent predictors of treatment response. Regression coefficients (B), odds ratios [Exp(B)], and 95% confidence intervals (CI) are shown

Logistic regression analysis including 194 patients

*B* Regression coefficient, *CI* Confidence interval, *Exp(B)* Odds ratio

## Discussion

This study examined the relationship between International Index of Erectile Function scores and Hounsfield unit values of the penile corpora cavernosa in patients diagnosed with erectile dysfunction, with the aim of exploring their association with treatment response. Our findings suggest that HU measurements may provide complementary radiological information that could contribute to the characterization of erectile dysfunction and its underlying vascular features.

Erectile dysfunction has multifactorial origins, including organic, psychogenic, and mixed etiologies, and therefore requires a multidisciplinary diagnostic and therapeutic approach. Although phosphodiesterase type 5 inhibitors represent a cornerstone of ED management, a substantial proportion of patients do not achieve adequate response. In this context, identifying objective parameters that may help contextualize treatment response remains clinically relevant [11].

Hounsfield unit measurements represent quantitative, CT-based radiodensity parameters that primarily reflect tissue composition rather than direct perfusion; however, in the context of erectile dysfunction, they may provide indirect information related to tissue characteristics influenced by vascular and metabolic factors. The spleen, owing to its rich vascularization, has been used in prior radiological studies as a pragmatic indicator of systemic perfusion, with lower splenic tissue density reported in conditions associated with systemic hypoperfusion such as diabetes mellitus and advanced age [3, 12]. Importantly, splenic tissue density does not constitute a validated measure of global microvascular function and was therefore used in this study as an adjunctive, contextual parameter rather than a reference standard.

Penile erection depends on adequate arterial inflow, intact veno-occlusive mechanisms, and functional smooth muscle, all of which are influenced by tissue perfusion. Lower penile HU values may be associated with alterations in microvascular characteristics, particularly in the presence of systemic risk factors such as diabetes mellitus, hypertension, and endothelial dysfunction. In this study, significant negative correlations were observed between post-treatment sexual satisfaction scores and HU values in selected penile segments. The observed paradoxical associations, including lower HU values in some treatment responders, may reflect early treatment-related hemodynamic changes such as vasodilation and increased intratissue fluid content rather than structural remodeling; however, these mechanisms cannot be established within the scope of this retrospective analysis.

Treatment outcomes further support a cautious interpretation of these findings. Daily tadalafil therapy was associated with significant improvements across all IIEF domains, consistent with previous reports [13–15]. Positive correlations between erectile function scores and left midshaft HU suggest that objective tissue density may be associated with patient-reported outcomes. Segmental variability in statistically significant findings may reflect heterogeneous vascular and anatomical characteristics across penile regions and warrants further investigation.

The observed correlations between splenic and penile HU values further support the concept that systemic vascular and metabolic factors may influence local penile tissue characteristics. Rather than serving as a standalone assessment tool, splenic HU measurements may provide complementary, contextual information when interpreted alongside penile measurements. Similarly, the internal consistency observed across different penile segments suggests that HU measurements reflect underlying tissue characteristics rather than random variability.

The International Index of Erectile Function remains a widely accepted instrument for the assessment of erectile dysfunction. Although inherently subjective, it captures multiple clinically relevant domains [16]. Integrating objective imaging-based parameters with patient-reported outcomes may contribute to a more comprehensive characterization of erectile dysfunction, without implying direct predictive capability. Given the vascular focus of the present study, erectile function was considered the primary domain of interest, whereas analyses involving other IIEF domains were exploratory and intended to provide contextual information rather than direct vascular assessment.

Prior studies have demonstrated that penile color Doppler ultrasonography findings are associated with response to phosphodiesterase type 5 inhibitor therapy, particularly in vasculogenic erectile dysfunction. In a cohort of men with ED, patients with preserved arterial flow showed better response rates to PDE5 inhibitor treatment, while diabetic status negatively influenced outcomes [17]. These observations underscore the importance of vascular assessment in ED and provide a framework for exploring complementary imaging approaches.

We also evaluated the relationships between HU values and various biochemical parameters. The negative correlation between fasting blood glucose and HbA1c levels with splenic HU supports prior evidence linking diabetes with microvascular compromise. These findings emphasize the close connection between erectile dysfunction and systemic diseases, particularly metabolic syndrome [18].

Hormonal associations were also observed. Positive correlations between PSA and penile HU may relate to prostate–penile vascular interplay. Negative correlations between penile HU and luteinizing hormone or prolactin underscore the influence of hormonal balance on erectile tissue. These findings are consistent with previous evidence indicating that systemic hormonal profiles, including testosterone levels, influence tissue composition and CT-derived radiodensity measurements [19].

Limited data are currently available regarding the application of CT-derived tissue density measurements in the evaluation of erectile dysfunction. A study by Yilmaz et al. demonstrated that HU measurements may aid in diagnostic evaluation and treatment planning in benign prostatic hyperplasia [20], supporting the broader applicability of HU-based analyses in urological conditions and highlighting the exploratory nature of the present study.

With respect to multivariable analysis, logistic regression did not identify penile or splenic tissue density parameters as independent predictors of treatment response. This finding does not negate the associations observed in univariate and correlational analyses, but rather reflects the multifactorial nature of erectile dysfunction, the retrospective design, and the exploratory scope of the study. Accordingly, HU measurements should not be interpreted as standalone predictors of treatment response, but as complementary parameters that may provide insight into vascular and metabolic influences relevant to erectile function.

From a clinical perspective, the present findings suggest that CT-derived Hounsfield unit measurements may provide complementary information when counseling patients with erectile dysfunction, particularly those with suspected vasculogenic etiology. Although HU values should not be used to predict treatment response on an individual basis, lower baseline penile tissue density may indicate underlying vascular or metabolic alterations that could influence responsiveness to phosphodiesterase type 5 inhibitor therapy. In this context, HU assessment may may assist clinicians in framing realistic expectations regarding treatment.

### Limitations of the study

This study has several limitations. Its retrospective, single-center design introduces potential selection bias, and most computed tomography scans were obtained for clinical indications unrelated to erectile dysfunction, raising concerns of indication bias. Although predefined inclusion and exclusion criteria were applied to reduce heterogeneity, residual bias cannot be fully excluded.

Another limitation is the absence of an a priori sample size calculation or power analysis. Owing to the retrospective design, the study population was determined by the availability of eligible patients with complete clinical and imaging data. In addition, the relatively short duration of tadalafil treatment may not fully capture long-term structural or microvascular changes in penile tissue. The lack of standardized protocols for penile Hounsfield unit measurement necessitated the use of segmental averaging, which may have introduced methodological variability.

An additional limitation relates to the use of splenic tissue density as a surrogate marker of systemic perfusion. Although the spleen has been employed in prior radiological studies as a pragmatic indicator of systemic hemodynamic and metabolic influences, splenic tissue density does not represent a validated or definitive measure of global microvascular function. Accordingly, splenic measurements were used in an adjunctive and contextual manner rather than as a reference standard. Finally, no formal adjustment for multiple comparisons was applied, increasing the risk of Type I error; therefore, all findings should be interpreted as exploratory and hypothesis-generating rather than confirmatory.

## Conclusion

Daily administration of tadalafil 5 mg was associated with significant improvements in International Index of Erectile Function scores and measurable changes in penile and splenic Hounsfield unit values on non-contrast computed tomography in patients with erectile dysfunction. These findings suggest that CT-based HU assessment may provide complementary objective information related to tissue characteristics and perfusion, particularly in patients with suspected vasculogenic erectile dysfunction. Future prospective, multicenter studies with standardized protocols and longer follow-up are required to validate the clinical utility of HU measurements in routine erectile dysfunction evaluation.

## Data Availability

Data are available from the corresponding author upon reasonable request.

## Declarations

### Use of artificial intelligence

Language editing and translation assistance from Turkish to English for selected sections of the manuscript (Abstract, Introduction, and Discussion) were provided with the assistance of ChatGPT (OpenAI, San Francisco, CA, USA). All scientific content, data interpretation, and final wording were critically reviewed and approved by the authors, who take full responsibility for the integrity of the work.

## Abbreviations

BMI: Body mass index
CI: Confidence interval
CT: Computed tomography
DM: Diabetes mellitus
ED: Erectile dysfunction
EF: Erectile function
FBG: Fasting blood glucose
FSH: Follicle—stimulating hormone
HbA1c: Hemoglobin A1c
HT: Hypertension
HU: Hounsfield unit
ICC: Intraclass correlation coefficient
IIEF: International Index of Erectile Function
IQR: Interquartile range
LH: Luteinizing hormone
NO: Nitric oxide
NPT: Nocturnal penile tumescence
PDE5i: Phosphodiesterase type 5 inhibitor
PRL: Prolactin
PSA: Prostate—specific antigen
ROI: Region of interest
SD: Standard deviation
SPSS: Statistical Package for the Social Sciences
TSH: Thyroid—stimulating hormone
TT: Total testosterone
